# Experiencia en la atención continua del cáncer de páncreas en un hospital de alta complejidad en Bogotá

**DOI:** 10.15446/rsap.V27n5.121373

**Published:** 2025-09-01

**Authors:** Samuel Rey-Robledo, Liliana Cuevas-López, Elio Fabio Sánchez

**Affiliations:** 1 SR: MD. Cirujano Oncólogo. M. Sc. Salud Pública. Pontificia Universidad Javeriana. Bogotá, Colombia. s.rey@javeriana.edu.co Pontificia Universidad Javeriana Salud Pública Pontificia Universidad Javeriana Bogotá Colombia s.rey@javeriana.edu.co; 2 LC: MD. Cirujana Oncóloga. M. Sc. Epidemiologia Clínica. Unidad de Cirugía Oncológica, Hospital Universitario San Ignacio. Bogotá, Colombia. lcuevas@husi.org.co Unidad de Cirugía Oncológica Hospital Universitario San Ignacio Bogotá Colombia lcuevas@husi.org.co; 3 ES: MD. Cirujano Oncólogo. Jefe de la Unidad de Cirugía Oncológica, Hospital Universitario San Ignacio, Departamento de Cirugía y Especialidades, Pontificia Universidad Javeriana. Bogotá, Colombia. efsanchez@husi.org.co Pontificia Universidad Javeriana Departamento de Cirugía y Especialidades Pontificia Universidad Javeriana Bogotá Colombia efsanchez@husi.org.co

**Keywords:** Carcinoma ductal pancreático, continuidad de la atención, Colombia, tratamientos *(fuente: DeCS*, *BIREME)*, Pancreatic ductal carcinoma, continuity of care, Colombia, treatments *(source: MeSH, NLM)*

## Abstract

**Objetivo:**

Describir y evaluar el continuo del cuidado de pacientes con adenocarcinoma ductal de páncreas (ADCP) en el Hospital Universitario San Ignacio (HUSI) entre 2017 y 2023.

**Métodos:**

Estudio observacional analítico de cohorte retrospectiva de pacientes mayores de 18 años con diagnóstico histológico de ADCP en el HUSI. Se recolectaron datos socio-demográficos, clínicos, de morbilidad, mortalidad y variables relacionadas con el continuo de atención. Se realizó un análisis descriptivo y bivariado utilizando pruebas de Chi cuadrado y Kruskal-Wallis.

**Resultados:**

Se incluyeron 105 pacientes. El 90,5% recibió tratamiento propuesto, principalmente paliativo (73,3%). Se encontró una alta atención fragmentada (AF) en el 41,9% de los pacientes. La AF se asoció significativamente con estar afiliado a una entidad administradora de planes de beneficio (EAPB) específica, haber tenido intención de tratamiento curativo y un estadio patológico temprano. El tiempo entre la cirugía y la adyuvancia fue mayor en pacientes con AF.

**Conclusiones:**

El estudio describe un tardío diagnóstico del ADCP, lo que condiciona un tratamiento con enfoque paliativo desde el inicio de la enfermedad. Hubo una alta prevalenia de AF, lo cual, aunado al diagnóstico tardío, sugiere la necesidad de mejorar la coordinación en la atención. Se identificó una asociación entre la AF y factores como la afiliación a determinadas EAPB, la intención de tratamiento curativo y el estadio patológico temprano, lo que resalta la importancia de intervenciones dirigidas a optimizar el acceso y la continuidad de la atención en esta población.

## Abstract (en)

**Objective:**

To describe and evaluate the care pathway of patients diagnosed with pancreatic ductal adenocarcinoma (PDAC) at Hospital Universitario San Ignacio (HUSI), between 2017-2023.

**Methods:**

An analytical, observational, retrospective cohort study was conducted. The study population included patients over 18 years of age with histopathological confirmed diagnosis of PDAC at HUSI. Data were collected on sociodemographics, clinical characteristics, morbidity, mortality, and variables related to the continuum of care. Descriptive and bivariate analyses were performed using Chi-square and Kruskal-Wallis tests.

**Results:**

A total of 105 patients were included in the study. The majority (90.5%) received the proposed treatment, with palliative care being the most frequent approach (73.3%). A high prevalence of care fragmentation (CF) was observed in 41.9% of patients. CF was significantly associated with affiliation to a specific Health Benefits Plan Administrator (EAPB), having the intention of curative treatment, and presenting with early-stage pathology. The time interval between surgery and adjuvant therapy was longer in patients with CF.

**Conclusions:**

The study identifies a pattern of late diagnosis of PDAC, which frequently leads to an initial palliative treatment approach. There was a high prevalence of CF which, coupled with late diagnosis, suggests the need to improve coordination of care. A significant association between CF and factors such as affiliation to particular EAPBs, the intention to receive curative treatment, and early-stage pathology was identified, highlighting the importance of targeted interventions aimed at optimizing access and continuity of care in this population.

El cáncer de páncreas representa un desafío de salud pública a nivel mundial. Según estimaciones recientes de la Agencia Internacional para la Investigación del Cáncer, del año 2022, la neoplasia pancreática es la sexta causa de mortalidad por cáncer 1. En Colombia, el cáncer de páncreas ocupa el undécimo lugar en incidencia, pero asciende al sexto en mortalidad, con 2812 nuevos casos anuales y 2573 defunciones en el mismo periodo 1.

El ADCP se clasifica según su grado de resecabili-dad en resecable, borderline resecable (BR), localmente avanzado y metastásico 2,3. Esta clasificación se basa en el grado de infiltración de estructuras vasculares alrededor del páncreas y la presencia de metástasis a distancia 4. El tratamiento actual del ADCP es multimodal e incluye cirugía (considerada la piedra angular en lesiones BR y resecables), a menudo complementada con quimioterapia adyuvante, y en algunos casos radioterapia 2,4. El manejo de las lesiones metastásicas o localmente avanzadas suele involucrar quimioterapia sistémica, de acuerdo con el estado funcional del paciente y las comorbilidades 2,4,5.

El cuidado del cáncer se concibe como un proceso continuo y complejo, que implica múltiples etapas de atención. Este "continuo de atención" se refiere a la secuencia de servicios que un paciente recibe durante su enfermedad 6. Hopstaken et al. 7 definen este continuo como la medida en que la atención sanitaria se experimenta como coherente, conectada y relevante para las necesidades del paciente, lo que lo convierte en un aspecto clave de la calidad asistencial.

En Colombia, el sistema de salud es mixto, combinando elementos públicos y privados para garantizar el acceso a la atención. Se estructura en dos regímenes principales: un régimen contributivo, financiado por trabajadores y empleadores, que ofrece una amplia gama de servicios, y un régimen subsidiado, financiado por el Estado, destinado a poblaciones vulnerables.

Aunque la Ley 100 de 1993 8 estableció el sistema general de seguridad social en salud en Colombia (SGSSS), con el objetivo de garantizar la cobertura universal, la atención integral del cáncer aún presenta desafíos. La responsabilidad de la atención se ha transferido a las EAPB, que gestionan contratos diversos y en ocasiones ineficientes con instituciones prestadoras de servicios de salud (IPS) 9,10. En este contexto, se desconoce el impacto en la continuidad de la atención en pacientes con ADCP. Este estudio pretende describir y evaluar el continuo de cuidado clínico del ADCP en un hospital de IV nivel en Bogotá, Colombia durante un periodo establecido. La identificación de patrones y el análisis del cumplimiento de los estándares de tratamiento en ADCP pueden permitir mejorar la toma de decisiones y optimizar el abordaje terapéutico de esta patología.

## METODOLOGÍA

Se realizó un estudio observacional analítico de cohorte retrospectiva, que incluyó pacientes mayores de 18 años, con diagnóstico histopatológico de ADCP, atendidos en el HUSI entre enero de 2017 y julio de 2023.

Los datos fueron extraídos del sistema de historia electrónica SAHI del HUSI. Se identificaron los casos a partir de los siguientes diagnósticos registrados: "tumor maligno de la cabeza del páncreas", "tumor maligno del cuerpo del páncreas", "tumor maligno de la cola del páncreas", "tumor maligno de otras partes de páncreas" y "tumor maligno del páncreas no especificado". Los datos se recopilaron y almacenaron en línea a través de un servidor seguro que ejecuta la aplicación web Research Electronic Data Capture (REDCap).

**Figura 1:**
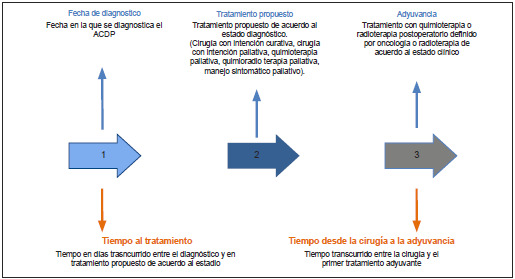
Continuo de la atención del adenocarcinoma pancreático (ADCP)

Se recolectaron variables sociodemográficas, clínicas basales, de morbilidad, mortalidad y relacionadas con el continuo de la atención del ADCP, según la definición operativa de sus componentes (Figura 1).

Se calculó el tiempo transcurrido desde el diagnóstico histológico hasta el inicio del primer tratamiento propuesto (neoadyuvancia, cirugía, quimioterapia paliativa, quimio-radioterapia paliativa o manejo paliativo de soporte). Adicionalmente, se determinó el tiempo transcurrido desde la cirugía hasta el inicio del manejo adyuvante en los pacientes sometidos a manejo quirúrgico con intención curativa. Se definió "atención fragmentada" como aquella que ocurre en más de una institución durante el continuo de la atención del ADCP 11,12. El análisis estadístico se realizó con el software R Studio (versión 2022.12.0+353).

Se calcularon medidas de estadística descriptiva: medianas y rangos intercuartílicos para variables continuas, y frecuencias y porcentajes para variables categóricas. Para explorar la asociación entre la variable AF y otras variables (EAPB, departamento de origen, estrato socioeconómico, estadificación tumoral, intención de tratamiento, tratamiento recibido, tiempo al tratamiento y muerte), se utilizó la prueba de Chi cuadrado. La asociación entre AF y el tiempo al tratamiento se evaluó con la prueba de Kruskal-Wallis.

De acuerdo con la Resolución 008430 de 1993 del Ministerio de Salud de Colombia, este estudio fue clasificado como "sin riesgo" y fue aprobado por el Comité de Ética e Investigaciones del HUSI.

## RESULTADOS

### Características sociodemográficas y clínicas

Se incluyeron 105 pacientes en la población de estudio, la mediana de edad fue de 66 años (RIQ=16 años), con una distribución según el sexo similar. La mayoría de los pacientes pertenecía a dos de las EAPB del SGSSS y tan solo dos pacientes tenían medicina prepagada. En cuanto al estrato socioeconómico, predominó el estrato III, aunque un 13,3% de los pacientes no tenía información registrada sobre su estrato. La mayoría de los pacientes residían en Bogotá (Tabla 1).

**Tabla 1:** Descripción de las características sociodemográficas y clínicas de la población de estudio

Variables	n=105	Total (100%)
Edad Mediana [Min, Max]	66	[29,85]
Sexo		
Masculino	56	(53)
Femenino	49	(47)
Departamento		
Bogotá	86	(81,9)
Cundinamarca	8	(7,6)
Boyacá	4	(3,8)
Tolima	3	(2,9)
Caldas	1	(0,9)
Casanare	1	(0,9)
Amazonas	1	(0,9)
Meta	1	(0,9)
EAPB *		
EAPB 1 (Régimen contributivo)	52	(49,5)
EAPB 2 (Régimen contributivo)	39	(37,1)
EAPB 3 (Régimen contributivo)	9	(8,6)
EAPB 4 (Régimen subsidiado)	2	(1,9)
EAPB 5 (Régimen contributivo)	1	(1)
Medicina prepagada	2	(1,9)
Estrato socio económico		
I	4	(3,8)
II	17	(16,2)
III	45	(42,9)
IV	18	(17,1)
V	6	(5,7)
VI	1	(1)
Rural sin dato	14	(13,3)
Clasificación de ASA*		
I	1	(0,9)
II	32	(30,5)
III	72	(68,6)
ECOG*		
I	71	(67,6)
II	27	(25,7)
III	7	(6,7)
Comorbilidad	72	(68,6)
IMC* Mediana [Min, Max]	22	[15, 38]
Resecabilidad		
Resecable	25	(23,8)
Borderline	3	(2,9)
Localmente avanzado	26	(24,8)
Metastásico	51	(48,6)
Estadio Clínico AJCC* 8ed		
I	4	(3,8)
II	16	(15,2)
III	34	(32,4)
IV	51	(48,6)
Estadio patológico AJCC* 8ed		
I	6	(5,7)
II	15	(14,3)
III	4	(3,8)
IV	1	(1)
No aplica	79	(75,2)

*Entidades Administradoras de Planes de Beneficios de salud (EAPB), American Society of Anesthesiologists (ASA), Eastern Cooperative Oncology Group (ECOG), Índice de Masa Corporal (IMC), American Joint Committee on Cancer (AJCC).

En relación con las características clínicas, la mayoría de los pacientes presentaron una clasificación según la American Society of Anesthesiologists (ASA) de III y un estado funcional según el Eastern Cooperative Oncology Group (ECOG) de I. Un 68,6% de la población tenía al menos una comorbilidad y la mediana de índice de masa corporal (IMC) fue de 22 (RIQ=4 Kg/im). Al momento del diagnóstico, el 48,6% de los pacientes presentaban enfermedad metastásica, mientras que un 23,8% se consideraban resecables y un 2,9% borderline resecable. De acuerdo con el estadio del American Joint Committee on Cancer (AJCC) 8.a edición, tanto en la clasificación clínica como en la patológica predominaron los estadios avanzados (Tabla 1).

### Continuo de la atención

Con respecto al tratamiento propuesto, la quimioterapia paliativa fue la opción más frecuente, seguida por la cirugía con intención curativa, que en su mayoría consistió en una pancreatoduodenectomía proximal. Los tratamientos paliativos incluyeron manejo sintomático, quimio-radio-terapia y cirugía, esta última realizada principalmente con derivaciones digestivas. El 1,9% de los pacientes recibió neoadyuvancia, la cual consistió en quimio-radioterapia. De estos pacientes, solo el 50% completó el esquema planeado de neoadyuvancia. Los dos pacientes que recibieron neoadyuvancia presentaron progresión de la enfermedad durante este tratamiento. En general, la mayoría de los pacientes recibió el tratamiento propuesto, y se encontró una proporción del 41,9% de atención fragmentada en la cohorte (Tabla 2).

**Tabla 2:** Intención de tratamiento en el momento del diagnóstico y desarrollo dentro del continuo de la atención de la población de estudio

Tratamiento propuesto	n=105	Total (100%)
Quimioterapia paliativa	49	46,7
Cirugía curativa	26	24,8
Pancreatoduodenectomía proximal	22	84,6
Pancreatectomia distal	4	15,4
Sintomático paliativo	17	16,2
Quimioradioterapia paliativa	6	5,7
Cirugía paliativa	5	4,8
Derivación digestiva paliativa	2	40
Derivación Bilio-entérica	1	20
Doble derivación	2	40
Neoadyuvancia	2	1,9
Tratamiento recibido	95	90,5
Atención fragmentada	44	41,9

Veintiuno de los veintiséis (80,8%) pacientes sometidos a cirugía con intención curativa recibió adyuvancia Predominó el uso de quimioterapia (95,2%, n=20), sobre la quimio-radioterapia (4,8%, n=t). El esquema adyuvante más frecuentemente utilizado fue folfirinox (47,6%, n=10), seguido por la combinación de capecitabina y gemcitabina (28,6%, n= 6), gemcitabina mono agente (14,3%, n=3) y cinco fluoracilo (4,8%, n=1). Un 76,2% (n=16) de los pacientes inició la adyuvancia de manera oportuna. Sin embargo, solo el 52,4% (11 de 21) completó la adyuvancia planeada, siendo la progresión de la enfermedad y la toxicidad limitante las principales razones para la interrupción (50%, n=5, en ambos casos). En un 28,6% (6 de 21) de los casos, la adyuvancia fue modificada debido a toxicidad limitante (83,3%, n=5) o progresión de la enfermedad (16,7%, n=1).

**Figura 2:**
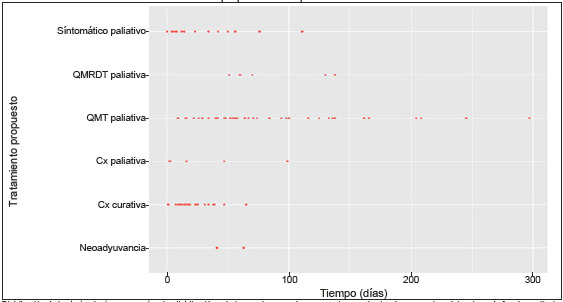
Dispersión del tiempo transcurrido entre el diagnóstico y el inicio del tratamiento propuesto de la población de estudio

La distribución de los tratamientos propuestos que recibieron los pacientes de la población de estudio se ilustra en la Figura 2. El análisis de los tiempos en el continuo de atención reveló variaciones significativas según el tipo de tratamiento. Los pacientes que recibieron neoadyuvancia presentaron una mediana de 52 días entre el diagnóstico y el inicio del tratamiento. El tiempo transcurrido hasta la cirugía con intención curativa tuvo una mediana de 18 días, y con intención paliativa una mediana de 16 días. Los tiempos hasta el inicio de la quimioterapia paliativa y la quimio-radioterapia paliativa tuvieron medias de 61 y 70 días, respectivamente. El inicio del manejo de mejor medidas de soporte paliativo tuvo una mediana de 23 días desde el diagnóstico. Con respecto al tiempo entre la cirugía curativa y el inicio de la adyuvancia, la mediana fue de 74 días. El 81% (n=85) de los pacientes falleció durante el periodo de seguimiento, y la causa más frecuente fue la progresión de la enfermedad en el 95,3% (n=81).

Distribución de los tratamientos propuestos. La distribución más homogénea se observa en el grupo de cirugía, cuyo valor mínimo logrado fue de un día. La mayor dispersión entre los valores extremos ocurrió en el grupo de quimioterapia paliativa, en ambos extremos estos pacientes presentaron AF.

### Morbilidad perioperatoria

Ningún paciente al que se le realizó cirugía paliativa presentó complicaciones. De los veintiséis pacientes llevados a cirugía curativa, se desconocieron los datos de complicaciones en nueve por ser operados en otra institución. De los quince pacientes restantes, seis presentaron al menos una complicación, de estas las más frecuentes fueron la fístula pancreática y la infección del sitio operatorio (ISO). Del total de las complicaciones, el 66,7% (n=4) fueron clasificadas como Clavien Dindo II y el 33,3% (n=2) III. No se presentaron reintervenciones o mortalidad postoperatoria.

### Variables asociadas a la fragmentación de la atención

Se encontró una asociación significativa entre la AF y estar afiliado a una EAPB específica (p=0,03). De igual forma, hubo una asociación entre AF y tener una intención de tratamiento curativo (p=0,03), observándose en el 61% de los pacientes esta intención; se encontró que la mediana del tiempo transcurrido entre la cirugía con intención curativa y el inicio de la adyuvancia fue mayor en pacientes con AF (81,5 días), en comparación con aquellos sin fragmentación (74 días). Adicionalmente, el estadio patológico se asoció con la AF (p=0,02), siendo más frecuente en pacientes con estadio I (100%). Por otro lado, no se encontraron asociaciones significativas entre la AF y el departamento de origen, el estrato socioeconómico, la recepción del tratamiento propuesto, la finalización de la adyuvancia y la mortalidad (Tabla 3).

**Tabla 3:** Análisis bivariado entre el desenlace de tener atención fragmentada y las variables de departamento, entidades administradoras de planes de beneficios, estrato, intención de tratamiento, tratamiento recibido, estadio patológico, adyuvancia completa y muerte de la población de estudio

Tratamiento propuesto	Si		No		Valor p
	n=44	41,9 %	n=61	58,1 %	
Departamento					0,64
Amazonas	0	0	1	100	
Bogotá D.C	35	41	51	59	
Boyacá	2	50	2	50	
Caldas	1	100	0	0	
Casanare	0	0	1	100	
Cundinamarca	5	63	3	38	
Meta	0	0	1	100	
Tolima	1	33	2	67	
EAPB*					
EAPB 1 (RC)*	22	42	30	58	0,03
EAPB 2 (RC)*	12	31	27	69	
EAPB 3 (RC)*	7	78	2	22	
EAPB 4 (RS)*	2	100	0	0	
EAPB 5 (RC)*	1	100	0	0	
Medicina prepagada	0	0	2	100	
Estrato socioeconómico					0,06
I al III	22	33	44	67	
IV al VI	14	56	11	44	
Rural sin dato	8	57	6	43	
Intención de Tratamiento					
Curativo	17	61	11	39	0,03 1
Paliativo	27	35	50	65	
Haber recibido tratamiento	40	42	55	58	
Estadio patológico					
I	6	100	0	0	0,02
II	8	53	7	47	
III	2	50	2	50	
IV	0	0	1	100	
Adyuvancia propuesta completada	7	64	4	36	0,08
Muerte	33	39	52	61	0,29

*Entidades Administradoras de Planes de Beneficios de salud (EAPB), Régimen contributivo (RC), Régimen subsidiado (RS).

El presente estudio, que caracterizó el continuo de la atención del ADCP en un hospital de Bogotá, Colombia, destaca aspectos críticos en el abordaje de esta enfermedad. El hallazgo principal es la alta prevalencia de AF (41,9% de la cohorte), lo cual, aunado al predominio de tratamientos paliativos (73,3%), sugiere un diagnóstico tardío y posibles deficiencias en la coordinación y la oportunidad de la atención en esta población. Este hallazgo resalta la necesidad de comprender la dinámica del continuo de atención del ADCP en el contexto colombiano.

Estos resultados se encuentran en línea con las expectativas iniciales del estudio, dada la complejidad del sistema de salud colombiano y las características agresivas del ADCP. La alta proporción de pacientes diagnosticados en estadios avanzados (48,6% metastásico) explica la priorización de la quimioterapia paliativa (46,7%), lo que a su vez indica la necesidad de estrategias de detección temprana más efectivas, problemática que persiste como foco de investigación. En consonancia con la literatura, (15-20%) 2,4,5, un 24,8% de los pacientes del presente estudio se consideraron resecables y por lo tanto se les propuso manejo quirúrgico con intención curativa. La alta tasa de AF fue un hallazgo inesperado, y destaca la necesidad de comprender a fondo los factores que contribuyen a esta problemática.

De acuerdo con la literatura sobre la continuidad de la atención en cáncer 13, nuestros hallazgos reafirman la importancia de una atención coordinada para optimizar los resultados clínicos. La asociación significativa entre la AF y la afiliación a determinadas EAPB, la intención de tratamiento curativo y el estadio patológico temprano sugieren la existencia de barreras especificas en el acceso y la coordinación de la atención. Estas barreras podrían estar relacionadas con las particularidades de cada EAPB y la complejidad de los tratamientos oncológicos. A diferencia de otros estudios 11, los resultados de esta investigación identifican factores específicos del contexto colombiano que influyen en la AF. Entre estos factores se destaca la variabilidad en el número de pacientes afiliados a las diferentes EAPB y las diferencias en los contratos que estas establecen con las instituciones prestadoras de servicios de salud (IPS), lo cual podría afectar la calidad y la disponibilidad de los servicios. En los pacientes con estadio patológico I sometidos a cirugía con intención curativa, la AF alcanzó el 100%, un hallazgo que podría explicarse por la necesidad de valoraciones de diversas especialidades durante el proceso de atención, lo que aumenta la probabilidad de fragmentación. Finalmente, la imposibilidad de obtener información sobre las complicaciones postoperatorias en nueve pacientes operados con AF, debido a que fueron atendidos en otra institución, evidencia cómo la fragmentación dificulta la recopilación de datos relevantes para evaluar la calidad de la atención.

Aunque no hubo una diferencia significativa en el tiempo transcurrido hasta el tratamiento propuesto entre los pacientes con y sin AF, la mediana del tiempo entre la cirugía y la adyuvancia en el subgrupo de pacientes con AF que recibieron adyuvancia fue de 81,5 días. Este valor supera tanto la mediana observada en los pacientes de este estudio que no presentaron AF (74 días) como las ventanas de tiempo óptimas para la adyuvancia reportadas en la literatura (69 días) 14. Estos resultados sugieren la necesidad de optimizar los tiempos en el continuo de atención para mejorar la efectividad de los tratamientos.

Es fundamental considerar que, si bien la cirugía representa la única opción curativa para el ADCP, este procedimiento ha evolucionado hacia un enfoque multidisciplinario en centros especializados 2,4. La presencia de complicaciones severas se ha asociado con una menor probabilidad de recibir tratamiento adyuvante, lo cual puede impactar negativamente en la supervivencia global 14,15. A diferencia de lo reportado en Estados Unidos, donde se ha documentado que la terapia adyuvante se omite en más del 58% de los pacientes sometidos a un primer abordaje quirúrgico 16, en nuestra cohorte el 80,8% recibió adyuvancia, lo que sugiere la viabilidad de esta secuencia terapéutica en el contexto del presente estudio.

Con respecto a la neoadyuvancia, si bien su uso fue limitado en nuestra cohorte (1,9%), su potencial para mejorar los resultados en pacientes con enfermedad BR es cada vez más reconocido 3,17,18. En este estudio, los dos pacientes que recibieron neoadyuvancia presentaron progresión de la enfermedad, lo que resalta la necesidad de una cuidadosa selección de los pacientes y una reevaluación constante durante el tratamiento neoadyuvante.

Las implicaciones de este trabajo son relevantes para el diseño de políticas y la práctica clínica. La necesidad de intervenciones dirigidas a mejorar la continuidad de la atención en el ADCP es evidente. Estas intervenciones podrían incluir la implementación de modelos de gestión de casos centrados en el paciente, el desarrollo de guías de práctica clínica integradas, el fortalecimiento de la comunicación entre los diferentes niveles de atención, y la evaluación de la eficiencia de los contratos entre las EAPB y las IPS. Asimismo, es fundamental promover el acceso a centros especializados, con equipos multidisciplinarios y experiencia en el manejo de esta compleja enfermedad.

El estudio presenta algunas limitaciones: en primer lugar, el diseño retrospectivo puede introducir sesgos de selección y medición; en segundo lugar, las características de la población estudiada limitan la extrapolación de los resultados a todas las instituciones del país; en tercer lugar, dado que el 41,9% de los pacientes presentaron AF, el seguimiento y la observación de desenlaces se vieron limitados, lo que impidió la realización de un análisis multivariado que permitiera establecer factores causales o asociados. No obstante, se destaca que este estudio exploratorio y descriptivo contribuye a comprender la realidad del continuo de atención del ADCP a nivel nacional y plantea interrogantes relevantes para futuras investigaciones.

En conclusión, este estudio proporciona información valiosa sobre el continuo de atención del ADCP en un hospital de alta complejidad en Bogotá, Colombia. Los resultados resaltan la necesidad de abordar la FA, optimizar los tiempos entre las diferentes etapas del tratamiento y promover el acceso a centros especializados, para mejorar la calidad de la atención y los resultados clínicos en esta población. Futuras investigaciones deberán centrarse en la identificación de estrategias efectivas para mejorar la coordinación y la continuidad de la atención, así como en la evaluación del impacto de estas estrategias en la supervivencia y la calidad de vida de los pacientes con ADCP.

Este estudio describe el diagnóstico tardío del ADCP lo que lleva a un enfoque paliativo inicial. A pesar de que la mayoría de los pacientes cumplieron con la intención de tratamiento propuesto, se destaca la necesidad imperante de abordar la FA del ADCP, especialmente en un contexto hospitalario de alto nivel en Colombia, como lo solicita el plan decenal para el control del cáncer en el país 19. Se enfatiza la importancia de reconsiderar las políticas de atención oncológica y promover investigaciones que profundicen en la comprensión del impacto de la fragmentación en el cuidado del paciente con cáncer, especialmente en aquellos casos en los cuales la intención es curativa y se ven afectados por esta situación. Asimismo, resalta la necesidad de implementación de políticas públicas que impulsen la atención integral del cáncer en centros multidisciplinarios, para mejorar la calidad y eficiencia del sistema de salud. Este enfoque integral es esencial para mejorar la calidad y la eficiencia del cuidado ofrecido a estos pacientes en el sistema sanitario del país ♣
